# GABA_A_ Receptor Subunit Composition Drives Its Sensitivity to the Insecticide Fipronil

**DOI:** 10.3389/fnins.2021.768466

**Published:** 2021-11-29

**Authors:** Zineb Soualah, Antoine Taly, Lucille Crespin, Ophélie Saulais, Daniel Henrion, Claire Legendre, Hélène Tricoire-Leignel, Christian Legros, César Mattei

**Affiliations:** ^1^Univ Angers, INSERM, CNRS, MITOVASC, Equipe CarMe, SFR ICAT, Angers, France; ^2^Laboratoire de Biochimie Théorique, CNRS, Université de Paris, UPR 9080, Paris, France; ^3^Institut de Biologie Physico-Chimique, Fondation Edmond de Rothschild, PSL Research University, Paris, France

**Keywords:** fipronil, GABA_A_ receptor, synaptic/extrasynaptic receptor, voltage-clamp recording, cerebellum

## Abstract

Fipronil (FPN) is a worldwide-used neurotoxic insecticide, targeting, and blocking GABA_A_ receptors (GABA_A_Rs). Beyond its efficiency on insect GABA_A_Rs, FPN causes neurotoxic effects in humans and mammals. Here, we investigated the mode of action of FPN on mammalian α6-containing GABA_A_Rs to understand its inhibitory effects on GABA-induced currents, as a function of the synaptic or extrasynaptic localization of GABA_A_Rs. We characterized the effects of FPN by electrophysiology using *Xenopus* oocytes which were microtransplanted with cerebellum membranes or injected with α6β3, α6β3γ2S (synaptic), and α6β3δ (extrasynaptic) cDNAs. At micromolar concentrations, FPN dose-dependently inhibited cerebellar GABA currents. FPN acts as a non-competitive antagonist on ternary receptors. Surprisingly, the inhibition of GABA-induced currents was partial for extra-synaptic (α6β3δ) and binary (α6β3) receptors, while synaptic α6β3γ2S receptors were fully blocked, indicating that the complementary γ or δ subunit participates in FPN-GABA_A_R interaction. FPN unexpectedly behaved as a positive modulator on β3 homopentamers. These data show that FPN action is driven by the subunit composition of GABA_A_Rs—highlighting the role of the complementary subunit—and thus their localization within a physiological synapse. We built a docking model of FPN on GABA_A_Rs, which reveals two putative binding sites. This is consistent with a double binding mode of FPN on GABA_A_Rs, possibly one being of high affinity and the other of low affinity. Physiologically, the γ/δ subunit incorporation drives its inhibitory level and has important significance for its toxicity on the mammalian nervous system, especially in acute exposure.

## Introduction

**FIGURE 1:**
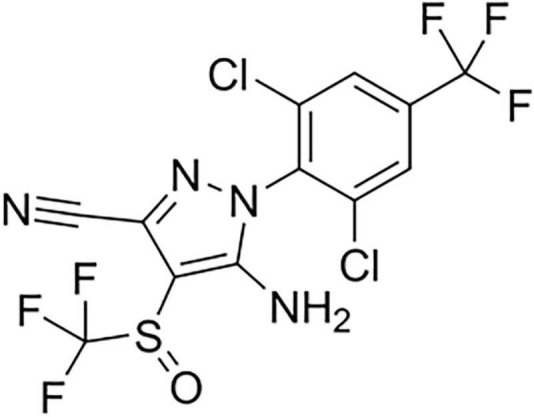
The 2D structure of fipronil (Pubchem CID: 3352).

Insecticides are used worldwide to increase crop yields or to fight vector-borne diseases. Restrictions to their use are due to insect resistances and off-target toxicity, including pollinators, mammals, and humans (Gibbons et al., 2015; Simon-Delso et al., 2015). Most insecticides target the nervous system, eliciting an overstimulation or a deadly inhibition of central or peripheral functions (Casida and Durkin, 2013). Of them stands fipronil (FPN, Figure 1), a phenylpyrazole molecule launched more than 30 years ago for pest control and known to act on GABA_A_ receptors (GABA_A_Rs) as a non-competitive antagonist or a negative allosteric modulator (Hosie et al., 1995; Ikeda et al., 2001). FPN binds to GABA_A_Rs, preferentially in the open state, thus promoting exacerbated excitability in the central and peripheral nervous system (Szegedi et al., 2005).

The efficiency of an insecticide relies on its ability to neutralize a pest at low concentrations without consequences on undesired targets. The lethal dose 50% (LD_50_) of FPN is typically 0.25 μg/g in house fly, and about 130 times less potent in mouse (Cole et al., 1993), suggesting a higher affinity for insect molecular targets. In addition, FPN has been shown to inhibit glutamate-chloride receptors, which are expressed only in invertebrates (Zhao et al., 2004). Although FPN was designed to selectively target insects, previous data have also proved it acts on vertebrate systems (Ikeda et al., 2001; Tingle et al., 2003). Acute human intoxication involving FPN revealed symptoms associated with the GABA transmission within the central nervous system, including seizure, agitation, and headache (Mohamed et al., 2004; Bharathraj et al., 2015). Confirming these biological signs in human, FPN induces hyperactivity, tremor, and seizure in mice (Cole et al., 1993). It has been more recently involved in memory impairment in rats, through its interaction with GABAergic networks (Godinho et al., 2016). Electrophysiological studies brought to light that FPN antagonizes mammalian GABA_A_Rs in native rat neurons or when expressed in heterologous systems by decreasing the opening frequency of the channel (Ikeda et al., 2001; Li and Akk, 2008). GABA_A_Rs are targeted by a collection of pharmacologically active molecules including anxiolytic, anesthetics, neurosteroids, and alcohol (Olsen and Sieghart, 2008) but little is known about their interactions with insecticides.

The ionotropic GABA_A_R is a heteropentameric protein incorporating five subunits in total (α1-6, β1-3, γ1-3, δ, ε, π, θ, ρ1-3) among which three are different (Sigel and Steinmann, 2012). The receptor functional stoichiometry requires 2α, 2β, and a third complementary subunit: most of GABA_A_Rs display ternary subunit arrangement, mainly αβγ and αβδ isoforms (Olsen and Sieghart, 2009). In brain GABAergic networks, synaptic GABA_A_Rs are localized in the postsynaptic neuronal membrane and mediate a fast, strong, and transient “phasic” neuronal inhibition preventing neuronal overexcitation (Farrant and Nusser, 2005). Extrasynaptic GABA_A_Rs are localized at the somatic, dendritic and axonal levels of the neuronal membranes, distant from the release sites of GABA and are responsible for a long-lasting, slow, weak, and constant “tonic” inhibition which modulates the post-synaptic response by influencing the overall rate of neuronal excitability, namely the action potential firing (Farrant and Nusser, 2005; Kullmann et al., 2005). The nature of the GABA_A_R subunits determines their cellular localization and consequently their participation in the phasic or tonic inhibition and their pharmacological properties. A large amount of data shows that the third and complementary subunit leads to a synaptic or extrasynaptic localization. When harboring the γ2 subunit, GABA_A_Rs are predominantly synaptic (except for α5β2/3γ2); whereas the δ subunit confers an extrasynaptic localization (Nusser et al., 1998). The α6 subunit can be associated either with the γ2 or the δ subunit and is expressed in granule cells in both synaptic and extrasynaptic localizations (Nusser et al., 1998). In fact, the α6 subunit is predominantly found associated with the δ subunit to form a functional receptor located in the cerebellum (Wisden et al., 1992). It is also present in sensory networks (Gutiérrez et al., 1996).

In heterologous systems, such as *Xenopus* oocyte, functional heteropentameric GABA_A_Rs are classically obtained by the co-expression of three different subunits: α and β, with γ or δ. Beside, binary αβ receptors lacking the third γ or δ subunit, also form functional entities in heterologous systems, and although their physiological function is still a matter of debate, they are expressed next to ternary receptors and should be considered as therapeutic targets with specific pharmacological and biophysical properties (Bencsits et al., 1999; Sieghart and Sperk, 2002; Mortensen and Smart, 2006; Che Has et al., 2016; Chiou et al., 2018). However, the co-expression of three αβγ/δ subunits univocally lead to a ternary GABA_A_Rs (Angelotti and Macdonald, 1993) and it is likely that native receptors are predominantly of ternary organization (Olsen and Sieghart, 2009).

Few studies have explored the mode of action of FPN on mammalian GABA_A_R. It has been shown that the GABA_A_R subunit composition is a key feature to explain the affinity and binding of FPN to its mammalian target (Ratra and Casida, 2001; Charon et al., 2011). The β3 subunit is proposed to contain the FPN biding site (Ratra et al., 2001). In this study, the inhibitory effects of FPN on cerebellum membranes and recombinant α6β3γ2S/δ GABA_A_Rs were investigated for the potential effects of this insecticide on phasic and tonic GABAergic inhibition. In addition, we challenged FPN with GABA_A_Rs without any complementary subunit, to highlight the putative third subunit-dependent effects, and on β3 homopentamers, since β3 is a structural cue in FPN/GABA_A_R interaction (Ratra et al., 2001). Finally, our results prompted us to explore the interaction between FPN and α6β3-containing GABA_A_Rs through a 3D model.

## Materials and Methods

### Drugs

GABA (Sigma, Saint-Quentin-Fallavier, France) was prepared in the standard oocyte solution (SOS, see composition hereafter) and FPN (Sigma, Saint-Quentin-Fallavier, France; Figure 1) was diluted in DMSO and then its concentration range was prepared in SOS medium. Picrotoxin (PTX—Sigma, Saint-Quentin-Fallavier, France) was firstly diluted in DMSO to a final concentration of 0.1%. 100 μM PTX was then prepared in SOS medium. Control experiments using DMSO (0.5%) were performed. Etifoxine (EFX hydrochloride, Biocodex, Gentilly, France) was dissolved in DMSO at a final concentration of 0.1%.

### Ethics Statements

All animal procedures were carried out in accordance with the European Community council directive 2010/63/EU for the care and use of laboratory animals and were approved by our local ethical committee (N°A49007002 for rats) in addition to the French Ministry of Agriculture (authorization APAFIS#19433-2019022511329240 and B49071 for *Xenopus*). The NC3R’s ARRIVE guidelines were followed in the conduct and reporting of all experiments using animals. Four rats were killed to prepare cerebellum membranes (in four independent experiments) to attenuate the effects of individual polymorphism in GABA_A_R microtransplantation experiments. For TEVC, the data were collected from oocytes collected from 12 distinct *Xenopus* females. In our animal facility, oocytes were collected twice a week, leading to the use of two different females. Each animal was reused after 9 weeks to allow a full recovery (healing and for animal welfare).

### Animal Care

Rats and *Xenopus laevis* females were used for cerebellum and oocytes preparations, respectively. Wistar rats of 200–250 g were obtained from the Animal Facility Centre of the Hospital/University of Angers. Rats were maintained with *ad libitum* access to standard diet and tap water and accommodated in individual cages under controlled conditions of room temperature and illumination (12 h light/dark cycle).

*Xenopus* oocytes were prepared as previously described (Mattei et al., 2019). Briefly, adult female *Xenopus laevis* were purchased from Centre de Ressources Biologiques Xénopes (Rennes, France) and were bred in the laboratory according to the recommendations of the Guide for the Care and Use of Laboratory Animals of the European Community. Oocytes were harvested from *Xenopus laevis* frogs under 0.15% tricaine anesthesia. All animals recovered within 2–3 h. Each female is operated every 3 months, not less and no more than 5 times.

### GABA_A_R Subunit Cloning

The cDNAs encoding the α6 and δ subunits used in this work were cloned in mouse as previously described (Mattei et al., 2019). pGW1 (= pRK5) plasmids containing cDNAs encoding mouse β3 and γ2S subunits were provided by Steven J. Moss (Department of Neuroscience, Tufts University, Boston, United States).

### Rat Membrane Preparation

We adapted the method of membrane transplantation described previously (Miledi et al., 2004). Adult Wistar rats (male and female) were euthanized with CO_2_ (5%) for 6 min. The brain and cerebellum were removed and stored at −80°C. Tissues were ground on ice with 200 mM glycine buffer (sucrose 300 mM—glycine 200 mM—NaCl 150 mM—EDTA 50 mM—EGTA 50 mM, supplemented with protease inhibitors). A portion of the homogenate was aliquoted, frozen in liquid nitrogen and stored at −80°C for use in the protein assay. After homogenization, the samples were centrifuged for 15 min at 9,500 g and 4°C (12132-H angular rotor, SIGMA2-16K centrifuge). The supernatant was recovered—a part was aliquoted for protein assay—and centrifuged for 2 h at 100,000 g and 4°C (MLA-130 angular rotor, Beckman Coulter OPTIMA MAX-XP ultracentrifuge). The new supernatant was aliquoted and stored at −80°C. The pellet, which contains membranes, was suspended with 10 μl glycine buffer 5 mM, and stored at −80°C. The membrane preparations can be used for protein assay or for microinjection. These living membranes, which carry the native α6-containing receptors, incorporate within the oocyte membrane, and the oocytes can be stimulated with GABA (Palma et al., 2005).

### Protein Quantification

We used the same method as described previously (Crespin et al., 2016). Briefly, the protein content was determined with a BCA™ Protein Assay Kit (Pierce^®^). Several dilutions were performed with samples to ensure detection in the range. Each dilution was injected twice in a 96-well plate. After incubation at 37°C, the plate was read at 570 nm using a plate reader (Multiscan Ascent Thermoscientific^®^). The protein content was calculated from a standard range of BSA (0–80 μM). The preparation of cerebellum rat membranes yielded a membrane suspension of 24 ng/nl protein concentration.

### Oocyte Preparation

Oocytes were harvested as previously described (Mattei et al., 2019). Briefly, oocytes were collected from female anesthetized *X. laevis* with tricaïn 0.15 M for 15 min and washed first in a solution of SOS (NaCl 100 mM, KCl 2 mM, MgCl_2_ 1 mM, CaCl_2_ 1.8 mM, HEPES 5 mM—pH 7,4), and then in a Ca^2+^-free SOS solution. They were incubated under gentle stirring with collagenase (2 mg/ml) and trypsin inhibitor (0.8 mg/ml) for 5–10 min and manually defolliculated. Then, oocytes were stored in SOS medium supplemented with antibiotics (gentamicin 0.04 mg/ml, penicillin/streptomycine/pyruvate 0.22 mg/ml) at 4°C.

### Membrane Microinjection and GABA_A_R cDNA Injection

**FIGURE 2:**
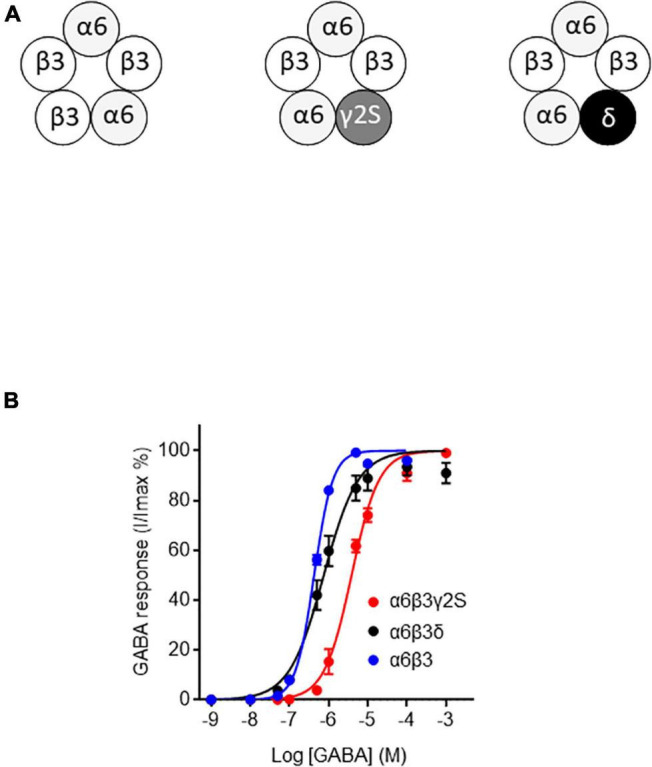
GABA sensitivity of ternary and binary α6-containing GABA_A_Rs. **(A)** Stoichiometric organization of α6-containing GABA_A_Rs used in this work (see Baumann et al., 2001; Baur et al., 2010; Sigel and Steinmann, 2012). **(B)** Concentration-response curves for α6β3γ2S, α6β3δ, and α6β3 GABA_A_Rs. Data were best fitted by non-linear regression to the Hill equation with variable slope α6β3γ2S: EC_50_ = 3.85 ± 0.28 μM and Hill-coefficient = 1.24 ± 0.08, *R*^2^ = 0.98; α6β3δ: EC_50_ = 0.75 ± 0.09 μM and Hill-coefficient = 0.99 ± 0.09, *R*^2^ = 0.95; α6β3: EC_50_ = 0.43 ± 0.01 μM and Hill-coefficient = 1.83 ± 0.09, *R*^2^ = 0.99). Data are mean ± SEM (*n* = 4–8) of at least two independent experiments.

Defolliculated stage V-VI oocytes were microinjected with rat cerebellum membranes or cDNA using a nano-automatic injector (Nanoject II, Drummond Scientific Company, Pennsylvania, United States). For optimal GABA-evoked current recordings (>10 nA), 55.2 nl of cerebellum membrane preparation corresponding to 1325 ng of proteins were injected by oocyte. The α6β3 (1:1), α6β3γ2S (1:1:5), α6β3δ (1:1:5) combinations were prepared at a concentration of 50 ng/μl for α and β, 250 ng/μl for γ2S, and δ to obtain. For each combination (Figure 2A), an amount of 450 pg cDNA was injected into the cell nucleus. Oocytes were incubated at 18°C and tested 24–48 h after injection. FPN was pre-applied for 45 s before any GABA application. To check ternary α6β3γ2S and α6β3δ GABA_A_Rs, and binary α6β3 GABA_A_Rs, controls were performed with GABA (5.10^–7^ M) before and after addition of Zn^2+^ (10 μM), which does not affect ternary receptors, and inhibits GABA-induced current through binary receptors (Supplementary Figure 1). Control experiments were performed with the antagonist PTX (100 μM) (Supplementary Figure 1).

### Electrophysiology

To monitor the activity of functional GABA_A_R responses in *Xenopus* oocytes, we used a standard two microelectrode voltage-clamp technique as described previously (Mattei et al., 2019). Glass microelectrodes were made with a DMZ Zeitz puller and exhibited a resistance of 0.5–1.5 MΩ. They were filled with an intracellular solution containing 1 M KCl/2 M K acetate. Each oocyte was continuously bathed in a recording chamber with the SOS solution. The resting membrane potential of injected oocytes was about −30 mV and, in the voltage-clamp configuration, the holding current (Ih) was about 50 nA for a holding potential of −60 mV. We only chose oocytes with a stable resting potential. The resting membrane potential was measured at the end of each experiment. FPN was diluted in the perfusion solution and then directly applied in the oocyte-containing chamber. 48 h after cDNA injection, oocytes were tested using a two-electrode voltage-clamp amplifier (TEV-200A, Dagan Corporation, Minneapolis, United States), at a holding potential of −60 mV. Data were acquired with a pCLAMP system (Digidata 1440 and pCLAMP 10.0 software from Axon Instruments). Experiments were performed at room temperature. Control experiments using DMSO (0.5%) were performed. No change in holding currents were observed, when non-injected or injected oocytes were perfused with SOS solution with 0.5% DMSO.

### Molecular Model Preparation and Docking

The models for the three receptors were prepared depending on the closest available template. The β3 homopentamer was directly based on the PDB structure 4COF. For α6β3 and α6β3γ2 heteropentamers, an additional homology modeling step was required and was based on the structure of the α1β3γ2 heteropentamer (6HUG). The sequences of the human α6, β3, and γ2 GABA_A_R subunits were aligned with those of the template using T-Coffee software (Notredame et al., 2000). The model was then prepared by homology modeling using Modeler version 9.19 software (Sali and Blundell, 1993) with default settings. One hundred models were prepared, and the best model, according to the Discrete Optimized Protein Energy function (DOPE), was selected. For the three models, side chains were improved with Scwrl4 (Krivov et al., 2009). The models were then evaluated with Molprobity and improvements on side chains were considered (Williams et al., 2018).

The docking has been performed with AutoDock Vina (Trott and Olson, 2010). The ligands and proteins were prepared with prepare_ligand4.py and prepare_receptor4.py scripts, respectively. The docking was restricted using a docking box of 15 A side, in the upper and lower binding sites identified previously (Charon et al., 2011). Figures were prepared with PyMOL (Schrödinger and DeLano, 2020).

### Data Analysis

In electrophysiology, the amplitude of each current response was expressed as a% of the response to GABA EC_50_. The EC_50_ and the Hill coefficient (nH) were determined by non-linear regression (Figure 2B) using the Langmuir equation with variable slope. We excluded data (i) in case of potential drift (>0.6 mV) after pulling out the electrodes from the oocytes and (ii) when current amplitudes were < 10 nA or > 2 μA. When required, current densities were calculated using membrane capacitance. GraphPad Prism 7.02 (GraphPad Software, San Diego, United States) was used for all graphs and statistical analyses. Normality of data distribution was validated using Shapiro-Wilk test to choose a parametric or a non-parametric test. Statistical significance tests between groups were performed using variance analysis (one-way ANOVA) followed by Tukey’s *post hoc* test for comparison of all groups or non-parametric Kruskal-Wallis procedure, followed by the *post hoc* Dunn’s test when appropriate. All data are presented as mean ± SEM of individual oocytes from at least two separate female *Xenopus*. Differences with *p* < 0.05 were considered significant (* for *p* < 0.05, ^**^ for *p* < 0.01, ^***^ for *p* < 0.001, ^****^ for *p* < 0.0001).

## Results

### Effect of Fipronil on Microtransplanted Rat Cerebella

**FIGURE 3:**
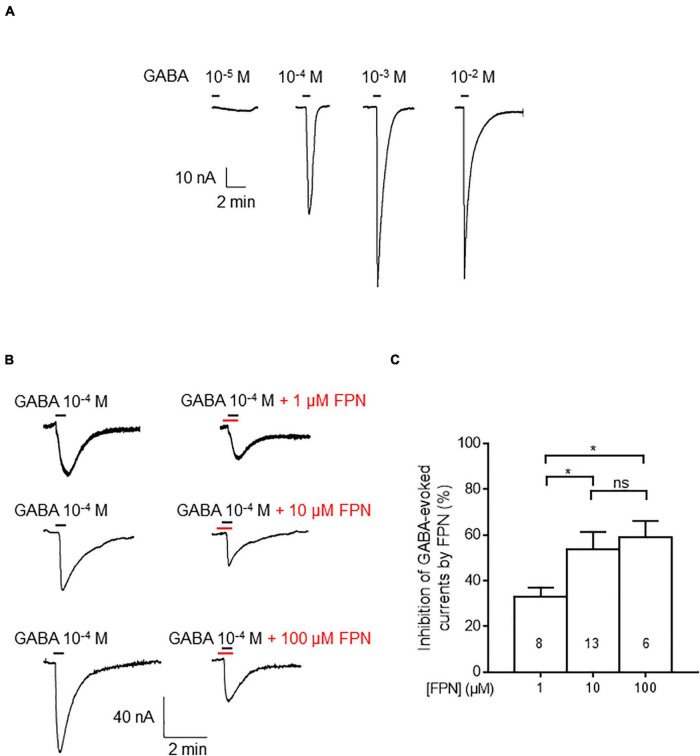
Inhibitory effects of FPN on GABA-induced currents in membrane-transplanted oocytes. **(A)** Representative traces of currents evoked by increasing concentrations of GABA in an oocyte transplanted with rat cerebellum membranes. **(B)** Effects of FPN (1, 10, 100 μM) on GABA-evoked currents. Control experiments denote the addition of 10^–4^ M of GABA (left), before simultaneous addition of GABA and FPN (right). **(C)** Histograms showing the concentration-dependent inhibitory effects of FPN on GABA-evoked currents elicited by oocyte transplantation with rat cerebellum membranes. The number of recorded oocytes is indicated inside the bars. Data are mean ± SEM. Multiple comparisons were performed using one-way ANOVA tests followed by Tukey’s *post hoc* correction (*p < 0.05, ns, not significant).

To assess the effect of FPN on native receptors embedded in their biological membranes, we used rat cerebella. In this context, GABA_A_Rs are fully functional as they can be activated by increasing concentrations of GABA (Figure 3A). We challenged microinjected oocytes with GABA (0.1 mM). FPN dose-dependently inhibited GABA-induced currents (Figure 3B). The inhibition level was 33.0 ± 3.9, 53.8 ± 7.5, and 58.8 ± 7.4% when GABA was applied with 1, 10 and 100 μM FPN, respectively (Figure 3C). Because the cerebellum mainly contains α6-, but also α1-, GABA_A_Rs (Wisden et al., 1992), we chose to express the ternary α6β3γ2S and α6β3δ receptors in *Xenopus* oocytes for investigating FPN putative antagonist activity and to decipher the role of the third subunit on its inhibitory effects.

### GABA Concentration Responses

To see if the FPN mode of action relies on the subunit composition, we first generated GABA concentration-response curves for the different GABA_A_R isoforms expressed in *Xenopus* oocytes, i.e., synaptic α6β3γ2S, extrasynaptic α6β3δ, and α6β3 (Figure 2A). For the synaptic α6β3γ2S, extrasynaptic α6β3δ and α6β3 GABA_A_Rs, we determined GABA EC_50_ values (Figure 2B) for subsequently evaluating FPN effects on each of these receptors. Our GABA EC_50_ values (see caption) are in accordance with data obtained by Mortensen et al. (2012), who transiently expressed various synaptic and extrasynaptic GABA_A_R in HEK-293 cells and reported a very similar overall ranking of GABA sensitivity. We obtained an EC_50_ of 0.75 μM for α6β3δ (Figure 2B), and Mortensen et al. (2012) reported EC_50_ of 0.17 μM for this same subunit combination. The EC_50_ was 3.85 μM for α6β3γ2S and 0.43 μM for α6β3.

### Antagonist Activity of Fipronil on Ternary Receptors

**FIGURE 4:**
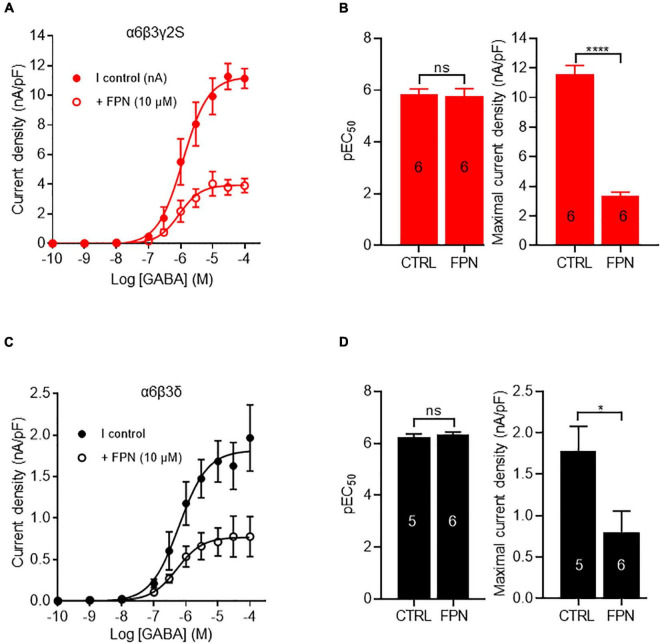
FPN effects on GABA-induced currents elicited by α6β3γ2S and α6β3δ GABA_A_Rs. **(A,C)** Concentration-response curves for α6β3γ2S and α6β3δ GABA_A_Rs. Data were best fitted by non-linear regression to the Hill equation with variable slope. **(B,D)** Comparison of EC_50_ and GABA-currents densities obtained with α6β3γ2S **(B)** and α6β3δ **(D)** GABA_A_Rs, in control (CTRL) and in the presence of FPN (10 μM). For α6β3γ2S, pEC_50_ was 5.83 ± 0.21 without FPN and 5.77 ± 0.28 with FPN; E_*max*_ was 11.6 ± 0.6 pA/pF without FPN and 3.4 ± 0.2 with FPN. For α6β3δ, pEC_50_ was 6.24 ± 0.12 without FPN and 6.33 ± 0.10 with FPN; E_*max*_ was 1.8 ± 0.3 pA/pF without FPN and 0.8 ± 0.2 with FPN. The number of recorded oocytes is indicated inside the bars. For both receptors, the normality of maximal current density distribution was validated using Shapiro-Wilk test and data were analyzed with unpaired *t*-test. EC_50_ values did not pass Shapiro-Wilk test and they were analyzed with non-parametric Mann and Whitney test (ns: non-significant, *p < 0.5, ****p < 0.0001. Data are mean ± SEM of at least two independent experiments (*n* = 5–6 cells) (see also Table 1).

To compare the inhibitory effects of FPN on extrasynaptic and synaptic receptors, we expressed ternary α6β3δ and α6β3γ2S combinations. FPN alone (10 μM) did not induce any current. We analyzed the effects of FPN (10 μM) with increasing concentrations of GABA (Figures 4A,C). For this purpose, we first applied 10^–9^ to 10^–4^ M GABA in the absence of FPN, which served as the control. This was followed by another set of experiments where the same concentrations of GABA were applied in the presence of 10 μM FPN. We measured the current density of each oocyte recorded. The concentration-response relationships for GABA in the absence and presence of FPN are shown in Figure 4. Our data show that the FPN effect is similar between the GABA_A_R combination: for both receptors, the addition of FPN did not modify EC_50_ values, whereas the maximal effect was significantly decreased (Figures 4B,D and Table 1). These observations agree with the fact that FPN behaves as a non-competitive antagonist (Li and Akk, 2008), which means that it targets an allosteric site on these two mammalian α6-containing receptors.

**TABLE 1:** GABA potency and efficacy on α6β3γ2S and α6β3δ GABA_A_Rs in the absence (CTRL) and the presence of 10 μM FPN.

	EC_50_ (μM, ± SEM)	pEC_50_ ± SEM	Hill coefficient ± SEM	Maximal current density (pA/pF ± SEM)	n
α*6*β 3γ 2S CTRL	2.7 ± 1.4	5.82 ± 0.21	1.1 ± 0.3	11.6 ± 0.6	6
+ 10 μM FPN	2.9 ± 1.5	5.77 ± 0.28	1.3 ± 0.5	3.4 ± 0.2	6
α*6*β 3δ CTRL	0.6 ± 0.2	6.24 ± 0.12	1.0 ± 0.3	1.8 ± 0.3	5
+ 10 μM FPN	0.5 ± 0.1	6.33 ± 0.1	1.1 ± 0.3	0.8 ± 0.3	6

### Inhibitory Effects of Fipronil on α6β3δ, α6β3γ2S, α6β3 GABA_A_Rs

**FIGURE 5:**
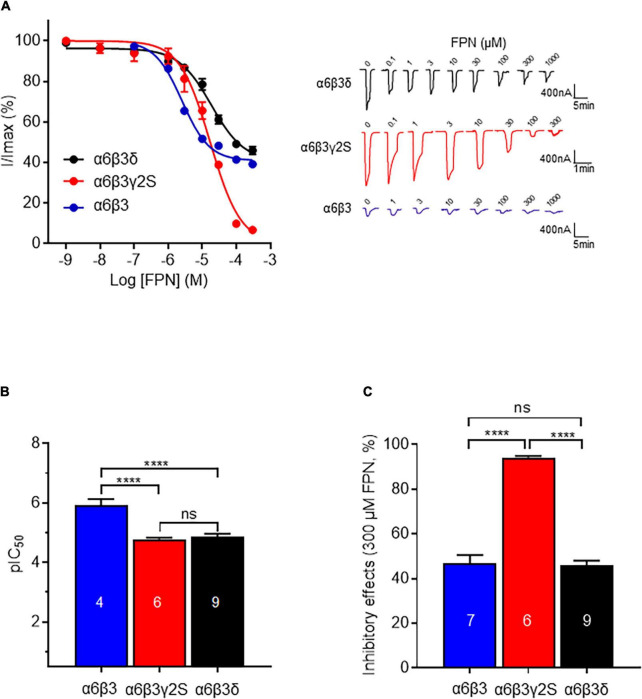
Comparison of inhibitory effects of FPN on α6β3γ2S, α6β3δ, and α6β3 GABA_A_Rs. **(A)** Left: Concentration-inhibition curves of FPN on α6β3γ2S, α6β3δ, and α6β3 GABA_A_Rs stimulated with GABA (EC_50_). Data were best fitted by non-linear regression to the Hill equation with variable slope. For α6β3γ2S, pIC_50_ was 4.86 ± 0.10 and Hill coefficient = 0.80 ± 0.05, *R*^2^ = 0.96; for α6β3δ, pIC_50_ was 4.78 ± 0.05 and Hill coefficient = 0.90 ± 0.12, *R*^2^ = 0.93; for α6β3, pIC_50_ was 5.93 ± 0.20 and Hill coefficient = 1.08 ± 0.29, *R*^2^ = 0.99. Right: Representative responses to concentration-inhibition of FPN on α6β3γ2S, α6β3δ, and α6β3 GABA_A_Rs stimulated with GABA (EC_50_). **(B,C)** Analysis of the inhibitory effects of FPN on α6β3γ2S, α6β3δ and α6β3 GABA_A_Rs. Potency (pIC_50_, **B**) and efficacy (maximum inhibition obtained with 300 μM FPN, **C**) of FPN on the three α6-containing GABA_A_Rs were compared. Normality of data distribution was validated using Shapiro-Wilk test and data were analyzed with one-way ANOVA followed by multiple comparison test with Tukey test (ns: non-significant, ****p < 0.0001). Data are mean ± SEM of at least two independent experiments (*n* = 4–9 cells) (see also Table 2).

To highlight the role of the complementary subunit in FPN-induced inhibition of GABA currents, we decided to challenge ternary and binary receptors. Oocytes injected with α6β3δ extrasynaptic ternary receptors were first subjected to GABA EC_50_ in the presence of increasing concentrations of FPN (Figure 5A). FPN (300 μM) produced a maximal inhibition of 46.0%, with an IC_50_ of 20.7 μM, which denotes a partial antagonist effect on extrasynaptic receptors (Table 2). To make sure that the current observed was mostly due to the ternary receptors (α6β3δ) and not binary receptors (α6β3), Zn^2+^-containing SOS was applied to inhibit GABA-evoked currents elicited by binary GABA_A_Rs, without altering GABA-evoked currents from ternary GABARs (Draguhn et al., 1990). The addition of ZnCl_2_ (10 μM) in a solution containing GABA 5.10^–7^ M triggered currents with similar amplitude to those obtained with the application of GABA alone (Supplementary Figure 1). The currents generated by the binary receptors were thus negligible, suggesting that most receptors expressed in the oocyte membrane were ternary α6β3γ2S. Furthermore, co-application of PTX (100 μM) and GABA (5.10^–7^ M) was performed to check if the observed currents were effectively GABA-driven. PTX induced an almost complete inhibition of current (Supplementary Figure 1).

**TABLE 2:** Fitting parameters of FPN inhibition on α6β3, α6β3γ2S, and α6β3δ GABA_A_Rs.

	IC_50_ (μM, ± SEM)	pIC_50_ ± SEM	Hill coefficient ± SEM	Maximal inhibition (% ± SEM)	n
α*6*β 3	2.4 ± 0.4	5.93 ± 0.20	1.08 ± 0.29	46.8 ± 3.7	7
α*6*β 3γ 2S	20.2 ± 1.7	4.86 ± 0.10	0.80 ± 0.05	94.1 ± 0.7	6
α*6*β 3δ	20.7 ± 2.6	4.78 ± 0.05	0.90 ± 0.12	46.0 ± 2.0	9

We then challenged the effect of FPN on α6β3γ2S synaptic GABA_A_Rs using the calculated EC_50_. FPN concentration-dependently antagonized α6β3γ2S (Figure 5A). Inhibition of the current was 94% with 300 μM FPN, and the calculated IC_50_ was 20.2 μM. This result shows a discrepancy in the inhibitory effect of FPN, as a function of the complementary subunit: on the one hand, IC_50_ values are similar between α6β3γ2S and α6β3δ; on the other hand, the inhibition is full for α6β3γ2S, and partial for α6β3δ. Again, to verify that the current recorded was due to the ternary α6β3γ2S receptors, we used Zn^2+^. The subsequent addition of ZnCl_2_ in the medium did not modify the GABA-induced current, while PTX (100 μM) inhibited these currents (Supplementary Figure 1).

To highlight the role of the complementary γ/δ subunit in FPN mode of action, we performed the previous experiment again using the binary α6β3 receptor. Representative current traces are shown in Figure 5B. Unexpectedly, FPN did not fully antagonize the α6β3-driven current. The IC_50_ is 2.4 μM—10-fold lower than ternary receptors—and the maximal inhibition is 46.8%, close to what is observed with α6β3δ receptors. Our data suggest that FPN differentially antagonizes GABA_A_Rs, as a function of their subunit composition, and the complementary subunit appears to play a crucial role in this inhibition. Altogether, the stoichiometry of these receptors drives their pharmacological properties toward the phenylpyrazole insecticide FPN, since the current inhibition was almost complete for synaptic receptors and partial for extrasynaptic and binary receptors.

### Effect of Fipronil on β3 Homopentamers

**FIGURE 6:**
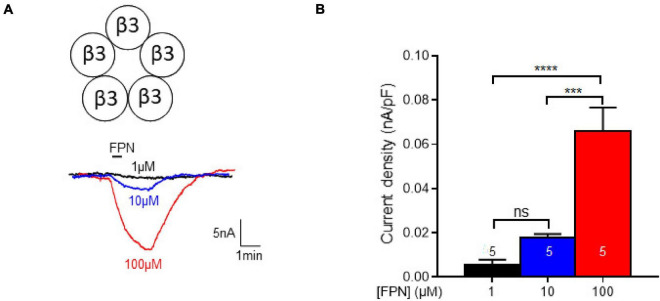
Effect of FPN on leak currents elicited by expression of β3-subunits in *Xenopus* oocytes. **(A)** Representative current traces obtained by increasing concentrations of FPN (1, 10, and 10 μM). **(B)** Analysis of FPN-induced currents. The current density increases as a function of FPN concentrations: it was 0.0058 ± 0.0021 nA/pF, 0.0177 ± 0.0018 nA/pF, 0.0661 ± 0.0105 nA/pF for 1, 10, and 100 μM FPN, respectively. The number of recorded oocytes is indicated inside the bars. Data are mean ± SEM of at least two independent experiments. Multiple comparisons were performed using one-way ANOVA tests followed by Tukey’s *post hoc* correction (***p < 0.001, ****p < 0.0001, ns, not significant).

The β3 subunit of mammalian GABA_A_Rs is of particular importance: although it has never been identified *in vivo*, it was the first GABA_A_R structure solved at high-resolution (Miller and Aricescu, 2014). This subunit has been shown to influence the binding of FPN to GABA_A_Rs (Ratra et al., 2001). The β3 homopentamer stands in an open conformation and proved to be insensitive to GABA or muscimol but can be positively modulated by pentobarbital (Wooltorton et al., 1997). The β3 leak currents could be inhibited by EFX (Supplementary Figure 1C), as it has been shown previously (Hamon et al., 2003). Then, this homopentamer was challenged to increasing concentrations of FPN which elicits dose-dependent currents (Figure 6). We can notice that these currents display small amplitudes (∼15 nA), compared to GABA-induced currents elicited by binary or ternary GABA_A_Rs (∼200–1,000 nA). In addition, FPN did not induce any current in oocytes expressing binary or ternary GABA_A_Rs, indicating that β3 homopentamers did not influence the GABA-mediated currents of Figures 2, 4.

Hence, FPN behaved as a pseudo-agonist of β3 GABA_A_Rs, after we have shown its role as an antagonist on binary and ternary receptors. This again highlights the versatile pharmacological properties of FPN on GABA_A_Rs, depending on their subunit composition, from non-competitive antagonist to positive allosteric modulator.

### Docking Model

**FIGURE 7:**
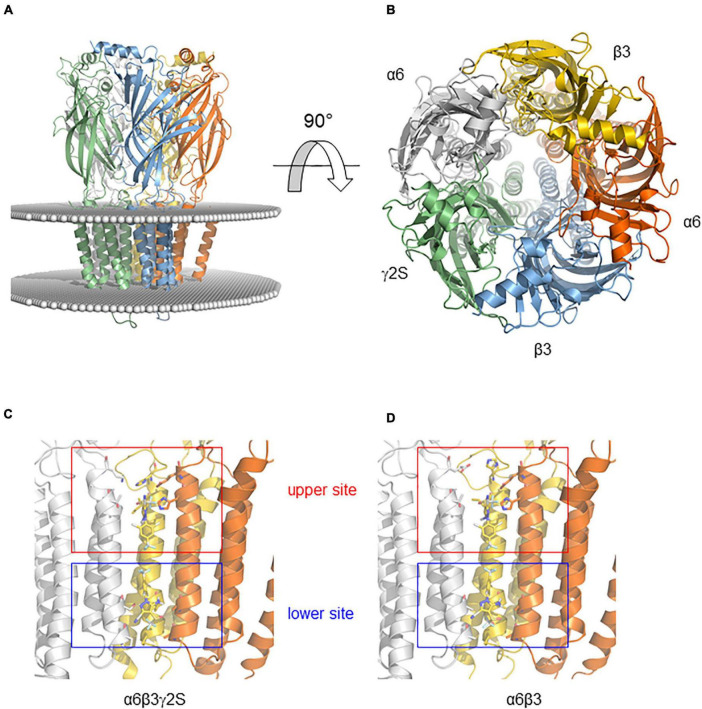
Binding modes of FPN obtained by docking on the mouse α6-containing GABA_A_R. **(A)** Model of the receptor viewed from the membrane plane. The protein is shown in cartoon representation with a different color code for each polypeptide. The position of the membrane is represented by a sphere positioned at the level of lipid head groups as determined by the Orientations of Proteins in Membranes database. **(B)** Model of the receptor viewed from above the membrane (rotation of 90° from **A**). **(C,D)** Binding modes of FPN obtained by docking on the mouse α6β3γ2S **(C)** and α6β3 **(D)** GABA_A_Rs. Close-up showing the FPN-binding pocket (FPN appears in sticks). FPN interacts with an upper site (Ser272)—nearby the extracellular part of the membrane—and the second site (Val257)—located near the intracellular part of the membrane.

We generated homology models of α6β3 and α6β3γ2S GABA_A_R to predict how FPN binds to their receptor site (Figure 7). The docking was guided by the prior knowledge that FPN is an open channel blocker and has two putative binding sites in the ion channel, lined by the M2 transmembrane segments (Perret et al., 1999; Charon et al., 2011). Indeed, our docking finds two binding modes, located nearby Val257 and Ser272 (Figures 7C,D). We reasoned that these two putative binding sites may explain the different pharmacological properties observed at Figure 5, because FPN appears in contact with residues that differ depending on the subunit (Figure 7). Both sites could be accessible to FPN and occupied simultaneously. The model presented is speculative and based on a possible docking. Consequently, our hypothesis deserves to be verified with mutagenesis experiments.

## Discussion

GABA_A_R antagonists, notably insecticides and convulsants, display differential activities at their site receptor just like GABA_A_R allosteric modulators, including anxiolytics and neurosteroids, which pharmacological properties depend on GABA_A_Rs subunit composition and regional expression in the brain, (Olsen, 2015). In this study, we looked at FPN effect on α6-harboring GABA_A_Rs with or without a complementary subunit. Binary (α6β3), synaptic (α6β3γ2S), and extrasynaptic (α6β3δ) receptors were expressed in *Xenopus* oocytes and challenged with FPN to measure the EC_50_ GABA-induced currents. We chose to work on the α6 subunit because it is exclusively expressed in the cerebellum, which function is dedicated to motor function in terms of movement, posture, and balance (Ghez and Fahn, 1985). Moreover, this subunit has been, among others, associated with genetic epilepsies, and might be considered as an interesting pharmaceutical target (Hernandez et al., 2011). On the other hand, the insecticide FPN is an antagonist of GABA_A_Rs and has been linked to different toxicological conditions in mammals, including seizures (Bharathraj et al., 2015; Gibbons et al., 2015). It has been shown in the past that FPN displays higher affinity toward the open conformation of the GABA_A_R (Ikeda et al., 2001). Once bound to FPN, the channel can still be targeted by GABA at the α/β interface, but it remains blocked due to the FPN binding. It displays high affinity for rat brain membranes with an IC_50_ value of 800 nM (Zhao and Casida, 2014). In mammals, FPN has been shown to compete with [^3^H]Ethynylbicycloorthobenzoate ([^3^H]EBOB) binding to man and mouse GABA_A_Rs with an IC_50_ of 942 and 1014 nM, respectively (Hainzl et al., 1998).

In our hands, FPN inhibits GABA-induced currents mediated by native GABA_A_Rs in cerebellum membranes or by heteropentameric GABA_A_Rs, with IC_50_ of 20 μM for ternary receptors and 2.4 μM for binary receptors. We noticed that FPN at 100 μM did not totally inhibit the GABA-induced currents in oocytes injected with cerebellum membranes. This could be due to the presence of GABA channels with a poor sensitivity to FPN. Alternatively, when tissue membranes are injected in oocytes, its plasma membrane can become unstable, which could explain what is observed at high concentrations of FPN.

The fact that subunit composition is a major contributor to FPN selectivity has been demonstrated in the past. The presence of the β3 subunit is a crucial feature in the interaction of FPN with GABA_A_R: binding and toxicity assays showed that β3 is part of the insecticide target and other subunits modulate the binding to confer selective toxicity (Ratra and Casida, 2001; Ratra et al., 2001). Competitive binding assay using human receptors have shown that FPN targets with high affinity β3 homopentamers (Ki 1.8 nM) and a decreasing affinity regarding the subunit composition: β3∼α6β3 > α6β3γ2, as indicated by the value of the IC_50_ (2.4, 3.1, 17 nM, respectively). Indeed, and as it has never been shown before, our data demonstrate that FPN dose-dependently activates β3 homopentamers, thus providing a confirmation of its direct interaction with this subunit, but as a positive modulator, rather than an antagonist. However, FPN elicits quite small currents in β3 subunit-injected oocytes and does not induce any current through ternary or binary receptors. Indeed, we cannot rule out the putative presence of β3 homopentamers in oocytes expressing heteropentamers, but with insignificant influence on the FPN antagonist effect.

This indicates that the molecular architecture of the GABA_A_Rs drives the selectivity of FPN and may be responsible for its toxicity. However, the limited inhibitory effect of FPN on both α6β3δ and α6β3 receptors prompted us to propose a model to explain this ambiguity. As it has been described previously, two putative binding sites have emerged for FPN, one being α1-Val257 close to the intracellular part of the membrane, the other α1-Ser272 close to the extracellular part, by docking FPN on the α1β2γ2 GABA_A_R (Charon et al., 2011). We can speculate that γ-containing ternary receptors could offer two binding modes to FPN, while in the α6β3δ and α6β3 receptors, the upper site (Ser272) would be favored, and the second site (Val257) might be less accessible and lowered. This discrepancy can explain the ability of FPN to bind binary and ternary α6-containing receptors, with a limited pharmacological inhibition of binary and extrasynaptic receptors.

FPN is known to bind with high-affinity the α6-containing binary GABA_A_Rs (α6β3) and with lower affinity the α6-containing ternary receptors (α6β3γ2S and α6β3δ) (Ratra and Casida, 2001). In contrast, as shown by our data, FPN is significantly more efficacious at ternary than binary receptors. Such pharmacological differences have been observed in the past, with various ligands. Recently, it has been shown that muscimol differentially activates binary and ternary GABA_A_Rs: co-expression of the δ subunit induced a greater sensitivity in α4β3-injected oocytes (Benkherouf et al., 2019). This result could be deducted from the observation that δKO mice exhibit reduced ^3^H-muscimol binding sites in the cerebellum (Mihalek et al., 1999). As demonstrated for muscimol, the binding properties of FPN depend on the γ2S subunit. Also, the benzodiazepine diazepam exhibits a greater efficacy on GABA currents when linked to ternary α1β2γ2 receptors compared to binary α1γ2 GABA_A_ receptors, and this might be explained by the higher binding site density at the ternary complex compared with the binary complex (Granja et al., 1997). This could explain a better accessibility of FPN to ternary receptors, by unmasking binding sites. More recently, the convulsant rodenticide tetramethylenedisulfotetramine (TETS) was investigated for its non-competitive antagonistic effect toward GABA_A_Rs (Pressly et al., 2018). TETS exhibit a clear receptor subtype selectivity: (i) it is more efficient at α2β3γ2l and α6β3γ2l receptors, and (ii) the complementary subunit appears to play a crucial role in this selectivity: TETS is 7 times more potent on ternary α2β3γ2l (IC_50_ 0.48 μM) than on binary α2β3 GABA_A_Rs (IC_50_ 3.37 μM).

The last feature illustrating the pharmacological versatility of FPN is its positive modulation on β3 homopentamers. These spontaneously open receptors have been shown to be positively modulated by pentobarbital, propofol and more surprisingly bicuculline, yet known as a competitive antagonist of ternary GABA_A_Rs (Wooltorton et al., 1997). Alongside, FPN proved to positively modulate murine β3 receptors in our study. This homopentamer, close to the rdl insect channels, has proved to exhibit high affinity for classical non-competitive antagonists, partly because of its symmetric organization (Chen et al., 2006). Our data raise the question of the binding site of FPN. As a non-competitive antagonist, FPN competes for the EBOB binding site on human β3 and α1β3γ2 GABA_A_Rs expressed in Sf9 cells, which highlighted the importance of the β3 subunit (Ratra et al., 2001). Two sites have already been observed for FPN, PTX and EBOB, one being a lower site (Val257) and the other an upper site (Ser272) (Charon et al., 2011).

## Conclusion

As predicted, FPN-induced toxicity in mammals may involve action at multiple receptor subtypes (Ratra et al., 2001). The results presented in our study have demonstrated the inhibitory modulation of the non-competitive FPN on GABA_A_ receptors depending on their subunit composition (α6β3δ, α6β3γ2S, and α6β3). These different combinations are either synaptic-like receptors (α6β3γ2S) or extra-synaptic (α6β3δ). The comparison of the FPN effects highlights the crucial and unexpected role of the third subunit in this inhibitory process. We show that ternary GABA_A_Rs composed of α6β3γ2S are totally antagonized by FPN while α6β3δ and binary α6β3-drivern GABA currents are only partially inhibited. Such inhibition levels have not already been reported because the subunit composition of GABA_A_R have not been characterized functionally with respect to their sensitivity to insecticides. Although there is a differential inhibition of FPN on synaptic and extrasynaptic receptors, both combinations proved to have their current blocked when expressed in *Xenopus* oocytes. It will be important to determine whether FPN and other GABA_A_R antagonists inhibit all binary and ternary receptor-mediated currents in a comparable way and if this could be related to physiological functions. The role of the third subunit deserves to be finely studied in other GABA_A_ receptors subjected to FPN. Site-directed mutagenesis on Ser272 and/or Val 257 will be helpful to validate the putative implication of both residues in ternary GABA_A_R allosteric modulation.

## Data Availability Statement

The original contributions presented in the study are included in the article/Supplementary Material, further inquiries can be directed to the corresponding author/s.

## Ethics Statement

The animal study was reviewed and approved by the local ethical committee (N°A49007002 for rats) in addition to the French Ministry of Agriculture (authorization APAFIS#19433-2019022511329240 and B49071 for Xenopus).

## Author Contributions

ZS, AT, OS, and LC conducted the experiments. AT, ChL, and CM analyzed the data. CM and ChL supervised the project and provided intellectual support for experimental procedures, data analysis. CM wrote the manuscript. HT-L, ChL, ClL, AT, and DH helped with manuscript writing. All authors read and approved the final manuscript.

## Conflict of Interest

The authors declare that the research was conducted in the absence of any commercial or financial relationships that could be construed as a potential conflict of interest.

## Publisher’s Note

All claims expressed in this article are solely those of the authors and do not necessarily represent those of their affiliated organizations, or those of the publisher, the editors and the reviewers. Any product that may be evaluated in this article, or claim that may be made by its manufacturer, is not guaranteed or endorsed by the publisher.
