# Supplementary figures and images for: GABAA Receptor Subunit Composition Drives Its Sensitivity to the Insecticide Fipronil
Source: Front Neurosci. 2021 Nov 29;15:768466. doi: 10.3389/fnins.2021.768466 (PMC8668240; doi:10.3389/fnins.2021.768466)

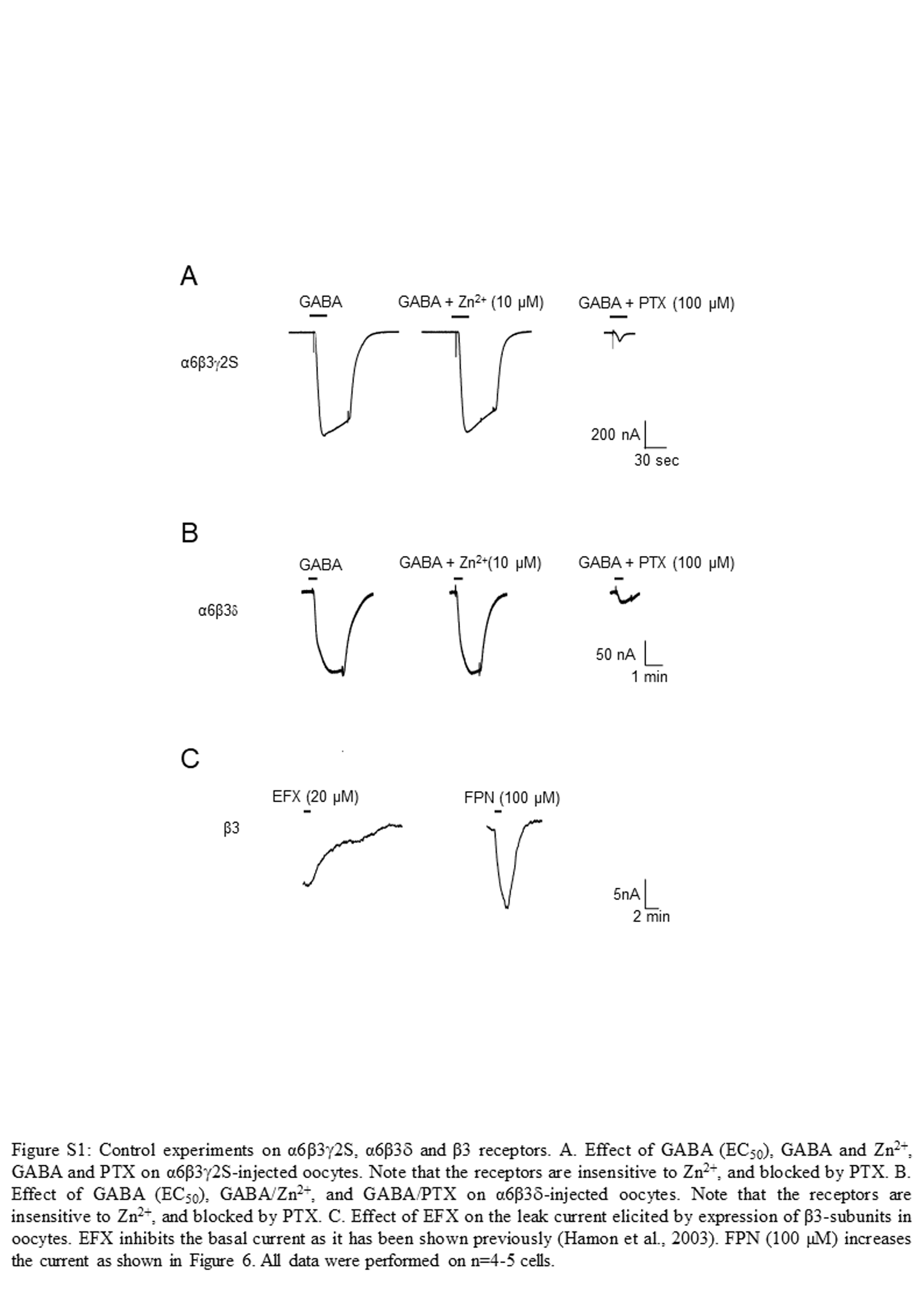

Supplement: Supplementary file 1 [file Image_1.TIF]
